# Designing in situ simulation in the emergency department: evaluating safety attitudes amongst physicians and nurses

**DOI:** 10.1186/s41077-017-0037-2

**Published:** 2017-02-08

**Authors:** Charlotte Paltved, Anders Thais Bjerregaard, Kristian Krogh, Jonas Juul Pedersen, Peter Musaeus

**Affiliations:** 1 Centre for Health Sciences Education, Aarhus University, Aarhus, Denmark; 2 Corporate HR MidtSim, the Central Region of Denmark, Aarhus, Denmark; 3 Department of Anaesthesia and Intensive Care, Aarhus University Hospital, Aarhus, Denmark; 4 CESU, Centre for Health Sciences Education, Aarhus University, Palle Juul Jensens Boulevard 82, Aarhus N, 8200 Aarhus, Denmark

**Keywords:** Education, Emergency medicine and first responders, Interprofessional collaborative practice, In situ simulation

## Abstract

**Background:**

This intervention study aimed to enhance patient safety attitudes through the design of an in situ simulation program based on a needs analysis involving thematic analysis of patient safety data and short-term ethnography. The study took place at an Emergency Department (ED) in the Central Region of Denmark. Research suggests that poor handover communication can increase the likelihood of critical incidents and adverse events in the ED. Furthermore, simulation is an effective strategy for training handover communication skills. Research is lacking, however, on how to use patient safety data and a needs analysis to the design of in situ simulation communication training.

**Methods:**

This is a prospective pre-post study investigating the interventional effects of in situ simulation. It used a three-pronged strategy: (1) thematic analysis of patient safety data consisting of reported critical incidents and adverse events, (2) a needs analysis based on short-term ethnography in the ED, and (3) pre-post evaluation using the validated Safety Attitudes Questionnaire (SAQ) and the Trainee Reactions Score.

**Results:**

Sixteen different healthcare teams participated composed by 9 physicians and 30 nurses. In the SAQ, participating staff scored their safety attitudes in six categories (*n* = 39). Two measures where significantly higher for the post-SAQ than those for the pre-SAQ: teamwork climate (*p* < 0.001) and safety climate (*p* < 0.05). The Trainee Reactions Score showed that the training was positively evaluated.

**Conclusions:**

This study designed a feasible strategy for implementing in situ simulation based on a needs analysis of critical incidents and adverse events and short-term ethnography.

**Electronic supplementary material:**

The online version of this article (doi:10.1186/s41077-017-0037-2) contains supplementary material, which is available to authorized users.

## Background

Inadequate communication can contribute to critical incidents (CIs) and adverse events (AEs) in the Emergency Department (ED) [1, 2]. As medical researchers and educators, we would like to know which kinds of inadequate communication are present in the studied ED organisation in order that we can tailor the training to the needs of the organisation. A needs analysis is a procedure to delineate challenges inherent in an organisation such as the ED in order to design training to ameliorate such issues [3]. However, very little published literature details how to perform a needs analysis in the ED [4] and how to use patient safety data and short-term ethnography in the analysis.

**Fig. 1:**
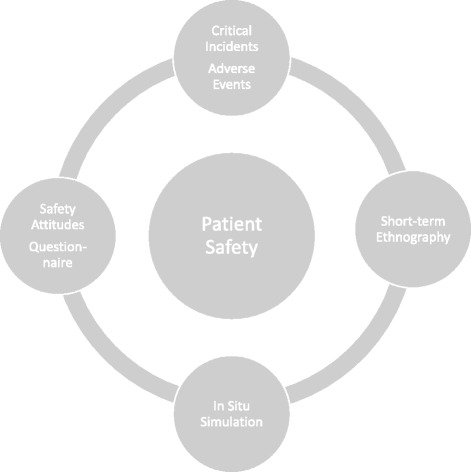
Overview of the study

Ethnography is an important qualitative methodological research and philosophical approach that researchers can use for needs analysis. The hallmark of conventional ethnography is participant observation where the researcher spends a long time in the field, sometimes up to several years. Most medical educational researchers may not have time to engage in long-time fieldwork due to publication pressures. Furthermore, practitioners working with simulation training might not have time for long-time fieldwork due to scheduling and production demands of their simulation training unit. These pressures make short-term fieldwork a more feasible strategy for needs analysis in order to ensure timely delivery of training.

Research seems lacking on how to conduct a needs analysis based on short-term ethnography. Short-term ethnography is a contemporary applied research approach designed to lead to informed interventions. In contrast to conventional ethnography, short-term ethnography is characterised by short-term field visits, sharpened focus, and a well-defined and rather narrow research question [5–7]. Furthermore, research is lacking on how a needs analysis can form the basis for design simulation training in the ED. The lack of such insight is a critical gap in aiming to develop efficient in situ simulation training programs to enhance patient safety at the ED. To sum up, a needs analysis is an important step in designing training, but it is unclear how to perform a needs analysis and use it for in situ simulation training of communication skills in the ED.

Why use in situ simulation to train communication skills for physicians and nurses in the ED? First, in situ simulation provides an opportunity to blend environments since it involves training with members of healthcare teams in their natural environment at the studied unit. This blending of learning and work environment may provide a feasible training strategy incorporating the complexity and resources found in the clinical environment. Second, while the literature supporting in situ simulation is relatively sparse, the evidence is promising and suggests that in situ simulation helps bridge the problem that clinicians might not be able to apply what they learn in actual work practice, because it was learned in a different site [8]. This is the problem of learning transfer defined as “using what was learned in one context to change performance in another” [9]. In situ simulation addresses the learning transfer problem at the level of teamwork skills when actual clinical teams practise together in the clinical setting where the team normally performs. Training at the workplace increases participants’ meaning-making and judgment that what they learn is relevant [10]. Another advantage with in situ simulation is the opportunity to schedule training times when people are at work and possibly able to train provided that they can break out in case of an emergency call. In addition, it can be difficult and costly to schedule time for clinicians to travel to the simulation centre to train [11]. Finally, research indicates that in situ simulation can be used to evaluate system competence and thus identify patient safety threats that are latent in the healthcare system or organisation [12].

Our stance is that in situ simulation might potentially utilize resources available at the ED by simulating the complex situations that the individual physician, nurse, or ED team faced. We argue that in situ simulation requires a needs analysis in order to tailor training to the needs of the studied ED. Present study investigates whether in situ simulation can enhance patient safety attitudes (Fig. 1).

## Methods

### Study design

This is a pre-post intervention study evaluating the effect of in situ simulation. We used a convergent parallel mixed method approach for data collection. We collected and analysed qualitative and quantitative data in parallel and merged the analysis at the final stage. The research question behind this study is: How can in situ simulation training informed by a needs analysis enhances patient safety attitudes in the ED? To answer this question, we used a three-pronged strategy of data generation:

1. Thematic analysis of CIs and AEs reported to the Danish Patient Safety Database.
2. Short-term ethnography as a means of needs analysis of the studied ED.
3. Evaluation of the program using the Safety Attitudes Questionnaire and the Trainee Reactions Score.

In this study, we administered the Safety Attitudes Questionnaire to evaluate participants’ benefits of an in situ simulation program based on (1) a thematic analysis of patient safety data (CIs and AEs) and (2) a needs analysis using short-term ethnography [13]. To evaluate the effect of the program, we used the validated Safety Attitudes Questionnaires (SAQ) [14]. The description of the in situ simulation program follows the guidelines published by Cheng et al. [15]. The setting was the ED at Aarhus University Hospital, one of the largest hospitals in Denmark and situated in the Central Region of Denmark with more than 40,000 emergency patients per year [16].

### Analysis of critical incidents and adverse events

Research has shown that communication errors between team members are the leading cause of CIs and AEs [17]. We aimed to inform in situ simulation scenarios based on a thematic analysis of CIs and AEs reported to the Danish Patient Safety Database in 2013. Our focus was only on the reports in the Central Region of Denmark. Two coders (JJ and KK) analysed the patient safety data using the qualitative software program NVivo 10. The coding was an iterative process, starting with a preliminary coding scheme that evolved through a continuous comparative approach. The preliminary coding scheme focused on the severity of the case, the healthcare professionals involved in the particular case, and the main problem described in subcategories like handover communication, or lack of knowledge and personnel. The two coders compared their scores and, in case of disagreement, reached an agreement by consensus amongst the whole research group.

### Short-term ethnography

To embrace the complexity of the ED setting and provide rich descriptions, we chose a qualitative research approach based on short-term ethnography. We observed and conducted informal interviews and administered questionnaires in the ED. Our roles as observers were inspired by what in anthropology is referred to as *direct moderate observation* informed by the backdrop of Spradley’s ethnographic approach to participant observation [18]. The data analysis was thematic and collaborative and involved a continuous theoretical dialogue between the research group members. For example, as the research project developed, both our data analysis and our reading of the literature on patient safety confirmed to us that handover communication would be an important field of investigation [19] and hence our observations focused on handover communication.

From June to November 2014, four trained observers (JJ, AB, KK, and SS) conducted 8 weeks of fieldwork comprising in total of 130 h cumulated for all observers in the ED. Two (JJ and AB) of the four observers were fifth year medical students trained as part time simulation operators in the simulation centre with 2 years experience. The third observer (KK) was an anaesthetic resident. The fourth (SS) was a project nurse. Both the resident and the project nurse were experienced simulation instructors and facilitators, both with more than 8 years of experience.

The observers wrote field notes in a pre-designed observation guide (Additional files 1 and 2) as cues, sentences, and direct quotes. During the fieldwork, the guide was re-designed to more specifically fit the complexity of the ED by, e.g. focusing on checklists such as the Situation Background Assessment Recommendation tool and an ABCDE-algorithm [20, 21].

Observers transcribed field notes immediately after each field visit adding additional comments and reflections afterwards to facilitate the analysis.

#### The in situ simulation program

The simulations took place in the ED in January–February of 2015 (Additional file 3) during the day shifts with 2 days of simulation each week and two 2 h training sessions each day (Additional files 4 and 5). Prior to the in situ simulation, the project nurse informed the staff about the program and all participating staff gave informed consent.

Based on the needs analysis and the training scenarios already in use at the MidtSim simulation centre, we developed three standardised medical scenarios (Additional files 4, 5 and 6): urosepsis, chronic obstructive pulmonary disease with exacerbation, and acute pancreatitis. Two medical experts read through, adjusted, and compared the scenarios in order to increase the clinical accuracy. We recruited three medical students and one nurse all with experience working with simulation to work as simulated patients. The project nurse (SS) instructed them on how to act their roles in the scenarios. To ensure realism, the make-up artist from the simulation centre worked with each simulated patient in each scenario. For instance, in the sepsis scenario (Additional file 6), the simulated patient had petechiae on the trunk and a clammy face made of Vaseline.

We aimed to form interprofessional teams. Twelve out of the 16 participating teams consisted of one or two physicians as well as two nurses. However, due to a lack of participating physicians, four teams consisted of two nurses that had the opportunity to call a physician for consultation. The simulated scenario was set in an ordinary equipped ED-single patient room. The project nurse was the main facilitator in all the scenarios and debriefings with assistance from the group. As mentioned, our needs analysis showed that handover communication was an important element to train and we did it by letting the participating nurses request assistance with patient care and at the end of each scenario where the leading physician initiated the transfer of the patient from the ED to the ward.

##### Evaluation

The Safety Attitudes Questionnaire and the Trainee Reactions Score

To evaluate the training effect for the participating physicians and nurses, we administered the Safety Attitudes Questionnaire (SAQ) developed by Sexton and Helmreich [14]. The SAQ is a validated questionnaire that measures attitudes and the effectiveness of interventions [14]. We used the SAQ in a pre- and post-training design [14]. The SAQ consisted of 36 items and these were answered using a five-point Likert scale as follows: disagree strongly = 1; disagree slightly = 2; neutral = 3; agree slightly = 4; and agree strongly = 5. There were six psychometric categories consisting of groups of 4–7 items: teamwork climate, safety climate, perceptions of management, job satisfaction, working conditions, and stress recognition. The items were adapted into Danish from the validated Norwegian version of the SAQ. Following modified principles from Beaton et al., three experts translated the Norwegian version and compared the translation to the original, back and forth until consensus was reached [22, 23]. To rate post-training reactions towards the in situ simulation program, we added the Trainee Reactions Score [24] consisting of nine items to the post-training SAQ. This score is collected from a questionnaire with a five-point Likert scale (Table 3). We used SPSS 21.0 for data analysis after completion of the in situ simulation program with the use of a paired-samples *t* test and the Wilcoxon signed-rank tests.

## Results

The Results section is organised in terms of the thematic analysis of CIs and AEs, a needs analysis that informed the in situ simulation program and finally evaluation of the program.

### Thematic analysis of critical incidents and adverse events

181,326 CIs and AEs were reported to the Danish Patient Safety Database in 2013. From these, we extracted 738 CIs and AEs related to EDs in the Central Region of Denmark. We excluded all cases of non-patient and non-team related CIs and AEs. We ended up with 36 cases that we coded as potentially fatal to the patient (Table 1).

**Tabl 1:**
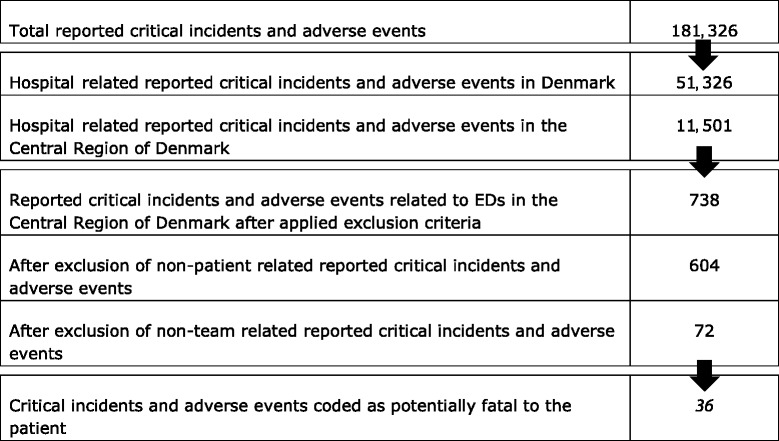
Critical incidents and adverse events in Denmark in 2013

We used thematic analysis to work inductively and deductively with codes from the material, which we subjected to membership checks [13]. The analysis of these 36 CIs and AEs focused on communication and handovers between team members and lack of knowledge or personnel. From the 36 reports coded as either severe or moderately severe to the patient, 21 (58%) reports described some sort of communication failure. For example, poor interdepartmental handover communication was coded as “severe”. The information in the CIs and AEs often confirmed that communication failed, but there were hardly ever descriptions or explanations about why a situation had gone wrong. We can illustrate this problem with a quote from a report coded as severe:

At 5 pm, we get a critically ill patient in the ED. [He presents with] a very high blood glucose, Kussmaul’s breathing, sepsis and pneumonia. […] At 7.15 pm, the attending medical physician schedules a transfer to the intensive care unit (ICU). We are told to wait half an hour before we go because they first have to relocate another patient. At 7.45 pm, we escort our patient to the ICU. At 7.54 pm, we return to the ED with our patient because the ICU wasn’t ready to take him in. They thought that the agreement was to call when they were ready for him.

This quote exemplifies the problem with interdepartmental communication and safe handover practice. The record emphasises two important features of the majority of the coded CIs and AEs. First, failure of communication is relatively common and can appear either directly reported as a medical error or described as a contributing factor in the series of events that led to a medical error. Second, the quote does not detail why or how it occurred that the ED was unable to take the patient. What were the thoughts and feelings, social interactions, etc. that lead to the outcome?

In short, we call for more detailed patient safety records if these records should form the basis of a needs analysis. This is not to say that patient safety data was useless in our case. The analysis of the CIs and AEs helped us confirm the relevance of the scenarios that we wrote based on the short-term ethnography and clinical experience. However, our contention is that it had been more clinically relevant and interesting if the analysis of the CIs and AEs had helped us throw vastly new insights into clinicians’ experiences and work practices and processes that lead to errors both at the individual, team, and organisational level [12, 25].

### A needs analysis that informed the in situ simulation program

The following two major themes emerged from our iterative reading and coding of the literature on handover and the inductive and deductive data analysis: handovers and the organisational design of the clinical encounter.

Clinical handover is defined as “the transfer of professional responsibility and accountability for some or all aspects of care for a patient or group of patients to another person or professional group on a temporary or permanent basis” [7].

In order to tailor the in situ simulation to the needs of the healthcare providers, we used short-term observation in the ED. Our role as health-care professionals or medical students who were doing participant observation gave us access to the department and we were able to stay at the ward during shifts, shadowing staff while they were doing their patient-related activities, and we wrote field notes about their ways of working, talking, and how they made sense of what they did. We selected pertinent field observations and asked key informants about how they interpreted them. The following results illustrate some of the observations relating to handover and the organisation of the clinical encounter.

During observations in the ED, we observed a sense of heightened urgency amongst professionals faced with interruptions. A frustrated nurse (A) declares:

To do a handover is despairing. We meet everyone who comes through the door to the ED, both the really ill patients, the outpatients, and those looking for the cafeteria!

We observed how team members struggled to create shared understanding in the team. We found that the healthcare providers agreed on the importance of handover. Despite this shared understanding, there was a lack of standardisation and consistency when handing over patients. Particularly, when patients were not critically ill or when professionals transferred patients between departments and from one section of the department to another. For instance, a coordinating nurse (B) says:

Typically, we start with the orthopaedic patients and then we talk about the most important patients first. There´s no particular way of doing it. It´s more like an unspoken agreement that we talk about the most important patients first.

The nurse speaks of an “unspoken agreement” about how to perform a safe and effective handover during a shift. Her point is that this requires a tacit shared understanding amongst her colleagues about how to prioritise patients. To accomplish this unspoken agreement, healthcare providers need a thorough grasp of norms in the ward.

We hypothesised based on the literature on systemic issues in healthcare that teamwork is important for successful clinical handover. We paid special attention to whether the health professionals adhered to guidelines for transferring patients in the ED including the use of SBAR (Situation Background Assessment Recommendation communication tool) [20, 26]. We found that staff used SBAR as a structured handover strategy only five times out of 22 handovers. After one observation without the use of SBAR, one researcher (AB) asked the physician why he did not use SBAR. His response was that he “did not always have the time” and “did not deem it necessary in none-life threatening situations”.

Through observation, we found that there was no designated place in the ED to deliver a safe handover to a colleague between shifts. Staff would sometimes hand over a patient in the hallway. The coordinating nurse would receive the patient and do the handover in front of the main counter in the ED with a huge risk of interruptions from other staff members, patients, or relatives. The lack of an organisational structure and physical space supporting handovers generated frustration and uncertainty in maintaining a professional role.

Nurse (C): “I actually find it kind of frustrating to have to wait for half an hour because I don’t know who will do the handover. I would feel so much better if I could just go into a room and get the report instead of waiting or looking for a nurse.”

Nurse (D):“It´s actually uncomfortable to do a handover in the corridors on the go.”

These quotes illustrated the importance of handover communication in the ED. The quotes and our reading of the literature led us to pursue three themes when writing training scenarios: (1) How can communication skills improve team members’ shared understanding? (2) How should team members avoid interruptions at the ward? (3) How do organisational factors affect team members’ handover communication?

Findings from the needs analysis showed that staff valued clear and structured communication and communication strategies such as thinking aloud in order to enhance patient safety. These communication skills improved shared understanding, but interruptions impaired communication. Furthermore, unstructured handover communication both interdepartmental and intradepartmental was putting patient safety at risk due to lack of organisational structures that were supposed to support such safe handover procedures.

### Evaluation of the in situ program using SAQ and Trainee Reactions Score

Originally, we planned 20 in situ simulation training sessions with 20 teams. However, due to cancellations, we were only able to conduct 16 training sessions, i.e. with 16 participating teams. A total of nine physicians and 30 nurses answered both the pre-SAQ and the post-SAQ. The overall response rate was 93% (39 out of 43).

We checked the six categories of the SAQ for outliers using SPSS 21.0. The safety climate category satisfied the criteria for normal distribution. The paired-samples *t* test for safety climate indicated that scores were significantly higher for the post-SAQ (Mean = 26.59, SD = 4.23) than those for the pre-SAQ (Mean = 25.74, SD = 4.41, *t*(38) = 3.451, *p* < 0.001). These results suggest that the in situ simulation program had a positive effect on the safety climate attitudes of physicians and nurses.

We performed the Wilcoxon signed-rank test on the related pre and post samples for the remaining categories. The tests indicated that the post-training teamwork climate scores (Mean = 20.6) were significantly higher than the pre-training scores (Mean = 19.9, *Z* = −1.961, *p* < 0.05). The results suggested a positive effect of the training on the teamwork climate at the ED. The remaining categories did not have a significant increase nor decrease after training (Table 2).

**Table 2:** Descriptive statistics

Variable (*n* = 39)	Pre (SD)	Post (SD)	Test	2-tailed *p* value
Safety climate	25.7 ± 4.41	26.6 ± 4.23	*t* = 3.45, df(14) Paired-samples *t* test	0.001
Teamwork climate	19.9 ± 1.78	20.6 ± 1.74	*z* = −1961, the Wilcoxon Signed-Rank Test	0.05

### The Trainee Reactions Score

Nine questions about trainee satisfaction delivered a snapshot of the general approval of the training and the content of it. Staff reacted overall positively to the training. All items scored on average between *strongly agree* (*5*) and *slightly agree* (*4*) on the Likert scale. The participating physicians and nurses indicated that the training was well organised (4.72 ± 0.61). Staff felt that they could use the strategies discussed during training and debriefing when returning to work (4.85 ± 0.366). In addition, staff reported that the in situ simulation training was an effective use of their time and that it could improve patient safety as shown below in Table 3.

**Table 3:** Trainee satisfaction

Item	Mean (*n* = 39)	Std. dev. (*n* = 39)
1) The training was well organised	4.72	.605
2) I am confident that I can perform the tasks that were trained	4.74	.442
3) I am confident that I understood the training content	4.90	.307
4) I am confident that I can use the knowledge that I learned on the job	4.85	.366
5) The training content was appropriate for my department	4.67	.772
6) Training will help my department improve patient safety	4.38	.711
7) As a result of this training, I feel more confident about my ability to work effectively in a team	4.36	.743
8) Training prepared me to work effectively in my job	4.21	.864
9) Training was an effective use of my time	4.87	.339

## Discussion

We have described the results of a pre-post intervention study that we had designed to create an effective in situ simulation program. In order to achieve this, we used a needs analysis of patient safety data of CIs and AEs in several hospitals and by a needs analysis using short-term ethnography at the studied ED.

The thematic analysis of the reported CIs and AEs lacked detailed information and rich descriptions about communication errors and in particular handover communication. This made our thematic analysis difficult when seeking to derive meaning from CIs and AEs. Specifically, the thematic analysis only shed little light on the role of team versus organisational processes generating CIs and AEs. The lesson for other researchers is that they should ascertain the level of detail in their national patient safety data records before they decide to conduct a needs analysis. Our experience from this study is that data codified at an abstract level e.g. with only one or more numbers (degree of severity, etc.) and data is not supplemented with qualitative descriptions it does not provide the details needed to conduct a needs analysis and gain inspiration for scenarios.

Several studies identify handovers as an area of high risk for CIs and AEs. However, handover research is diverse and not conclusive as to standardised handoff sheets [1, 27]. In a Danish study, root cause reports were analysed to describe communication failures between staff. In line with the results of our study, Rabøl et al. [28] found that the aspects of handover communication were particularly risky when there were no procedures for the handover and when patients were transferred between departments.

We found that short-term ethnography was a fruitful research strategy to inform the design of the in situ simulation program. Critics call short-term ethnography a watered-down version of conventional ethnography [29]. In advocating short-term ethnography, we accept at the outset the impossibility of gathering a complete, rich, and detailed understanding of the setting. However, we console somewhat in the fact that our team had intimate knowledge of the ED setting since team members were insider observers with high degree of background knowledge in the ED. Our aim in using a short-term ethnography was to balance the concept of insider observation from classical ethnography and concepts of experimental variation and intervention from psychology [5]. Through collaboration with staff in the clinical encounter, the intensity of this research became part of the way we learned about their clinical practice as we honed in on what was important to the participants. We learned more about the complexity of the ED from seeing how a phenomenon like handovers emerged in situ and in real time. The duration of ethnographic fieldwork should depend on the intensity and not per se time; i.e. if the approach is strategic and triangulated (interactive observations, interviews, theoretical perspectives), this is a feasible research strategy.

Research on in situ simulation is burgeoning as a method for delivering continuing education to healthcare providers training in their own clinical environment [8]. The main argument for simulation-based education (whether in situ or in the laboratory) is that it provides a safe place to learn from mistakes [30]. We suggest that for research and practice of in situ simulation in health care to advance, insights could be gained from the organisational sciences where there is increasing appreciation that training should be conceived of as emergent [31]. We argue that in situ simulation is a dynamic process not merely a training scenario enacted at the clinical ward followed by a static debriefing. In our study, this means that patient safety attitudes do not suddenly appear in the minds of the healthcare providers but emerge over time as a result of physicians and nurses contributing to the safety culture [32, 33]. Furthermore, safety attitudes are not separate from, but interwoven with physicians’ and nurses’ knowledge and skills about patient safety and team communication. Thus, safety attitudes could be an emergent phenomenon which as argued by Kozlowski et al. requires time before it moves from the level of physicians’ and nurses’ knowledge and attitudes to become fully manifests at the level of the collective (health organisation, team, ED) [31]. Since in situ simulation training takes place situated in the organisation where skills trained is later to be used, it is per definition embedded in how the training develops and this is a core consideration for explaining emergence, e.g. of team safety attitudes.

Effects of in situ simulation programs are likely underreported due to it being a new educational strategy with lack of intervention methods and research studies on the area [8]. In this study, the training effect was measured using the SAQ [14] and the results showed a significant increase in staff’s attitudes towards safety and teamwork climate. This suggests a positive effect of creating an in situ simulation program based on this. Research has shown a positive relationship between safety climate and patient safety. However, the evidence to support the effectiveness of intervention strategies to improve safety culture is limited and results differ [32, 33]. In an educational intervention study yet not involving simulation in the OR, Bleakley et al. also found significant improvement in the post-intervention SAQ teamwork climate scores [34]. However, Cooper et al. reported no effect on patient safety climate after a simulation-based training program in anaesthesia [35].

Based on the Trainee Reactions Score [24], the training was well received by staff and the scores also suggest that the learned communication skills could be transferred to clinical practice. Other studies have shown in situ simulation being effective at detecting system issues regardless of the clinical encounter. Guise and Mladenovic [36] conducted a multicentre intervention study in obstetric emergencies to test whether in situ simulation improved care and patient safety across geographically, organisationally, and clinically diverse hospital settings. They found the most common problem to be communication issues, which correlates to our study. Recurring in situ simulation can be a method to improve clinical care due to experiential learning experiences that provide a better opportunity to transfer skills into the real life of an ED setting. However, in a post-intervention survey, Patterson et al. pointed to that 77% of the healthcare providers reported little or no clinical impact despite the running of in situ simulations was for more than 1 year [12].

### Strengths and limitations

Strength of this study is the three-pronged and triangulated strategy. Gathering information from different sources added to the reliability of the data and to a better understanding of the complexity in the ED concerning handover and a way to identify staff’s specific training needs.

A limitation is that this study did not include a follow-up period to document any sustained positive achievement in teamwork and safety climate. We were not able to do a follow-up because 3 months into the study staff started implementing weekly in situ simulation. This could be an information bias but might also speak to the value of our study in bringing in situ simulation to the attention of staff at the ED.

Comparing the participating group with a control group in the simulation centre could prove a valuable addition. The results are significant; however, due to the relative low number of participating physicians and nurses (*n* = 39) and short training period (6 weeks), the effect of the intervention is not clear. Furthermore, it is a potential ethics issue when studying relatively few participants at a single site to ensure anonymity of participants. Without a doubt, we kept our analysis of participants’ records anonymous. However, participants might have felt that if we wanted, it would have been easy to identify their responses. We find it hard to judge whether this problem was exacerbated by the fact that many of us were employed at the same hospital as the participants. Did this make participants more at ease with us because we were perceived as friendly observers or more alert because we were wrongly perceived as spying observers whose reports could go to the management? Participants’ responses that they were highly satisfied with the intervention suggest that we were perceived as friendly observers and we were welcomed as professionals helping them become better.

To sum up, our study is a single-site study of only one ED in Denmark. However, we find that the strength of our study lies within the rigor of the intervention research methods employed, the systematic approach taken in trying to design an approach to needs analysis, and the triangulated means of data collection and mixed method approach to analysing data.

## Conclusions

The results of this study imply that an in situ simulation program can act as a significant catalyst for improvement in emergency staff’s safety and teamwork attitudes that might correlate with a more positive patient safety culture. We suggest a longitudinal study designed to investigate the impact of recurring team training in the ED and the evolving safety attitudes emerging over time.

## Additional files

Additional file 1: Observation chart. (PDF 1003 kb)

Additional file 2: Handover and re-evaluation chart. (PDF 918 kb)

Additional file 3: Calendar. (PDF 17.9 kb)

Additional file 4: Scenario overview. (DOCX 36 kb)

Additional file 5: In situ plan. (PDF 1566 kb)

Additional file 6: Urosepsis scenario. (DOC 83 kb)
