# Supplementary material for: Designing in situ simulation in the emergency department: evaluating safety attitudes amongst physicians and nurses
Source: Adv Simul (Lond). 2017 Feb 8;2:4. doi: 10.1186/s41077-017-0037-2 (PMC5806390; doi:10.1186/s41077-017-0037-2)
Supplement: Supplementary file 1 — Observation chart. (PDF 1003 kb) [file 41077_2017_37_MOESM1_ESM.pdf]

27/10

OBSERVATIONSSKEMA ECXCELLENT TEAMPERFORMANCE Musaeus, Krogh, Bjerregaard, Slot, Paltved & Juul (udfyld 1 pr. patient)

Teammedlemmer (Sæt kryds hver gang en ny person er til stede. Skriv gerne navn.)

Sygeplejerske 1 x Maria	Sygeplejerske 2	Sygeplejerske 3	Bioanalytiker	Serviceassistent 1	Serviceassistent 2	Lægesekretær	Ambulanceredder 1 X
Medicinsk forvagt	Medicinsk bagvagt	Kirurgisk forvagt	Kirurgisk bagvagt	Akutlæge X Lars	Anæstesiolog	Anæ.sygeplejerske	Ambulanceredder 2 X

Triagering (Sæt kryds ved triagefarve. Ved ændring, da en pil over til den nye farve)

Gul 18⁰⁰ ←	Orange X	Rød	Traume

Tidl "scannet" for et år siden uden forsnævring af kranspulsåre. men ej [illegible]

Tid	Sted: Sk. st. med / VAS 4-5	Obs. start: 17²⁸	Obs. slut:

Overordnet patientproblematik: Klassisk Angi billede ♂ 43 år, symptom blevet noget bedre siden debut omkring kl. 12⁰⁰. Stråle → hals/kraveben + v. arm. Intet [illegible] los ug. negv.

Hvorfra kommer sygehistorie (journal, patient, Falck etc.): Tidl. kendt med samme. Forsøgt NTG uden virkning.

Patientens vitale parametre: A: Fri B: Fri, let hyperventil. C: 120/90 P 78 SaO2 98 SR, EKG i.a.

D: Vågen, relevant. pt. 'bange'! "opistotonus" tilstand E:

Overskrift og oversigt, højdepunkter, begivenheder som er særligt vigtige. Skriv gerne et tema.

Handlebange ♂-pt, ♂, 43 årig, tidl verificeret coronar forsnævring. Nu angina, men alle "tests" inkl. ekg = i.a. Mangelfuld kommunikation → med. fv. ellers i stor træk fin kommunikation.

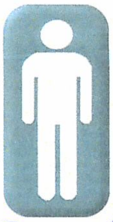

Kommunikeres præcist, relevant og kortfattet? (closed loops, hand-off, call-outs). Italesættes "mavefornemmelse", tentativ diagnose og symptomatologi? Tager modtager medansvar for sikker kommunikation?

18³⁷ Sgpl. kontakt til med.fv = noget "rodet" overlevering. (Sgpl. er helt ny) med fv ordinerer 5 mg morfin.

18⁴² Sgpl kontakt → akutlæge. Fin kort besked efter ISBAR og ABC principper. dog ej italesættelse af de to sidste = A og R

18⁴⁴ Sgpl → NTG spray: m. nogen effekt.

18⁴⁷ Sgpl laver "Morfin-closed-loop" med akutlæge

18⁴⁸ Akutlæge: skal vi tage den der "hurtigtest" Sgpl. nej den er taget. 9 var negativ.

Hurtigtest = Cobas test / TNT projekt.

Ikke af følelse [illegible] sgpl

Blev ABCDE brugt?

Airway	
Breathing	
Circulation	
Disability	
Exposure	

Blev ISBAR brugt? Følte SGPL

**Identifikation**	
Meddelers navn, funktion og afdeling	
Patientens navn, CPR	X
**Situation**	
Beskrivelse af situationen	X
Målte værdier	
Observerede ændringer i værdier	X
**Baggrund**	
Indlæggelsesdiagnose og dato	X
Kort sygehistorie	X
**Analyse**	
Vurdering af problemet	
**Råd**	
Giv eller bed om råd til videre behandling	

Nej, italesætter ej symptomer.

Fortolkning: Udfyld næste side, hvis der er tid efter beskrivelser ovenfor!

18⁵⁵ Ændret fokus på Coronar problematik → gul. Sgpl 9 læge laver plan for obs. [illegible]
