# Supplementary material for: Designing in situ simulation in the emergency department: evaluating safety attitudes amongst physicians and nurses
Source: Adv Simul (Lond). 2017 Feb 8;2:4. doi: 10.1186/s41077-017-0037-2 (PMC5806390; doi:10.1186/s41077-017-0037-2)
Supplement: Supplementary file 2 — Handover and re-evaluation chart. (PDF 918 kb) [file 41077_2017_37_MOESM2_ESM.pdf]

Debt
Problemer med egg. → login

Akut journal?
ubeskyttet Sex → Journal
thermofeeler → ?
fragering
urinprøve-farve
urinprøve → kønssygdom

billede foran
Skærm
Lægenotat

Han tog Rigtig Medicin?

Seddel til Martin MHT transcrip! har du den?

Handover and re-evaluation
In Situ Simulation 2015

**Scenario:**			
STAFF: Spl, Spl, læge, akutlæge			Date: 7/1-15
Department:	Instructors:	Observer:	Page:

For each level of re-evaluations and handovers :

- Identify any structured approach (ABCDE, ISBAR)
- Identify a new or altered situation
- Re-evaluate the effect of treatment
- Adjust course of action

QUALITY OF HANDOVERS AND RE-EVALUATIONS: CHECK ALL THAT APPLY

Example	Describe	Time
Scenario starts<br>Vital signs monitoring<br>Handover	Attention<br>RF 20<br>SAT 93<br>BP 90/85<br>P 122<br>Temp 39,3<br>opsum – SP<br>opsum – læge	
Vital signs<br>Handover	-Sat væske<br>+10 min → opsummering ⊕ (BT, SAT, ild; HF: ?)<br>Ringer til læge → angiver tilstand, Aktuelt Værdier, ↱ læge A-gas + Spørgsmål D+R	
Diagnosing<br>Treatment<br>Handover	A-gas – D<br>læge Ankommer / A, B, C, D → Hvad er gjort → VAS<br>~~9.50~~ 9.35	
Re-evaluate<br>Handover	9. ~~40~~ 35 → Journal optagelse<br>Closed loop → EKG → Jeg finder → Okay<br>A-gas → 9.41<br>Pamol i AB<br>→ 9.44 → opstart reeval indirekte i AB<br>Closed loop →	
Develop your diagnosis<br>Handover	→ diagnose?<br>→ RTG, overflytning<br>→ 18.00 → US<br>→ 1800 → Sp → Sepsis<br>~~ops~~ opsum<br>23 min, Plan.<br>Plan	
Finalizing<br>Conclude your actions<br>Handover	26 min → overlevering AKUT 2<br>rtg thorax inden overflytning<br>Er indlæggelsesgrund, Aktuelt<br>nu smerter<br>Blærebetændelse tidl.<br>feber 39,2°<br>Værdi: BT, P, EKG normalt	

C ✓ D
B ✓ E
A ✓
tidl.?
A-gas → gennemgår lagt katerer
Andet du skal vide!
