# Supplementary material for: Designing in situ simulation in the emergency department: evaluating safety attitudes amongst physicians and nurses
Source: Adv Simul (Lond). 2017 Feb 8;2:4. doi: 10.1186/s41077-017-0037-2 (PMC5806390; doi:10.1186/s41077-017-0037-2)
Supplement: Supplementary file 3 — Calendar. (PDF 17.9 kb) [file 41077_2017_37_MOESM3_ESM.pdf]

In Situ Sim

14/15

Oktober	November	December	Januar	Februar	Marts
On 1	Lø 1	Ma 1 In Situ test + prætest 49	To 1 Nytårsdag	Sø 1	Sø 1
To 2	Sø 2	Ti 2 In Situ test	Fr 2	Ma 2 6	Ma 2 10
Fr 3	Ma 3 OBS + Interview 45	On 3 Se Minna, In Situ NBG	Lø 3	Ti 3	Ti 3
Lø 4	Ti 4 OBS + Interview	To 4 Med ekspert	Sø 4	On 4 IN SITU	On 4
Sø 5	On 5 OBS + Interview	Fr 5 Med ekspert	Ma 5 Temadag 2	To 5 IN SITU	To 5
Ma 6 41	To 6 OBS + Interview	Lø 6	Ti 6	Fr 6	Fr 6
Ti 7	Fr 7 OBS + Interview	Sø 7	On 7 IN SITU	Lø 7	Lø 7
On 8	Lø 8	Ma 8 Med ekspert 50	To 8 IN SITU	Sø 8	Sø 8
To 9	Sø 9	Ti 9 1.deadline prætest Rette scenarier	Fr 9	Ma 9 7	Ma 9 11
Fr 10	Ma 10 OBS + Interview 46	On 10	Lø 10	Ti 10	Ti 10
Lø 11	Ti 11 OBS + Interview	To 11	Sø 11	On 11	On 11
Sø 12	On 12 OBS + Interview	Fr 12	Ma 12 3	To 12	To 12
Ma 13 42	To 13 OBS + Iw + Minna 9.30-11	Lø 13	Ti 13	Fr 13	Fr 13
Ti 14	Fr 14 OBS + Interview	Sø 14	On 14 IN SITU	Lø 14	Lø 14
On 15	Lø 15	Ma 15 Rykke prætest 51	To 15 IN SITU	Sø 15	Sø 15
To 16	Sø 16	Ti 16	Fr 16	Ma 16 8	Ma 16 12
Fr 17	Ma 17 Interview 47	On 17 2.deadline prætest	Lø 17	Ti 17	Ti 17
Lø 18	Ti 18 Interview	To 18	Sø 18	On 18	On 18
Sø 19	On 19 Interview	Fr 19	Ma 19 4	To 19	To 19
Ma 20 43	To 20 Interview	Lø 20	Ti 20	Fr 20	Fr 20
Ti 21	Fr 21 Interview	Sø 21	On 21 IN SITU	Lø 21	Lø 21
On 22	Lø 22	Ma 22 52	To 22 IN SITU	Sø 22	Sø 22
To 23	Sø 23	Ti 23	Fr 23	Ma 23 9	Ma 23 13
Fr 24	Ma 24 Analyse 48	On 24	Lø 24	Ti 24	Ti 24
Lø 25	Ti 25 Analyse	To 25 Juledag	Sø 25	On 25	On 25
Sø 26	On 26 Analyse	Fr 26 2. juledag	Ma 26 5	To 26	To 26
Ma 27 OBS + Interview 44	To 27 Analyse	Lø 27	Ti 27	Fr 27	Fr 27
Ti 28 OBS + Interview	Fr 28 Scenariskriv m Minna	Sø 28	On 28 IN SITU	Lø 28	Lø 28
On 29 OBS + Interview	Lø 29	Ma 29 1	To 29 IN SITU		Sø 29 Palmesøndag
To 30 OBS + Interview	Sø 30	Ti 30	Fr 30		Ma 30 14
Fr 31 OBS + Interview		On 31	Lø 31		Ti 31
23 arbejdsdage ekskl. 4 lørdage	20 arbejdsdage ekskl. 5 lørdage	21 arbejdsdage ekskl. 4 lørdage	21 arbejdsdage ekskl. 5 lørdage	20 arbejdsdage ekskl. 4 lørdage	22 arbejdsdage ekskl. 4 lørdage
