# Supplementary material for: Designing in situ simulation in the emergency department: evaluating safety attitudes amongst physicians and nurses
Source: Adv Simul (Lond). 2017 Feb 8;2:4. doi: 10.1186/s41077-017-0037-2 (PMC5806390; doi:10.1186/s41077-017-0037-2)
Supplement: Supplementary file 4 — Scenario overview. (DOCX 36 kb) [file 41077_2017_37_MOESM4_ESM.docx]

|  | | | | | |  |  |
| --- | --- | --- | --- | --- | --- | --- | --- |
| **Uge** | **Ugedag** | **Skadestue** **formiddag** | **Akut 1 torsdag eftermiddag** | **Akut 2 onsdag eftermiddag** | **Berørte specialer i tidsrummet/personalebehov**  **Læger deltage uforstyrret i debriefingen, derfor backup!** | |  |
| **2** | Onsdag 7.1.2015 | **Sepsis** |  | **Sepsis** | Medicin: 9 – 11 + 12.30 – 14.30 = 1 læge + 1 backup  Akutlæge: 9 – 11 + 12.30 – 14.30 = 1 læge + 1 backup  Sk.st sgpl: 9 – 11 = 2 sgpl  Akut 2: 12.30 – 14.30 = 2 sgpl | |  |
|  | Torsdag 8.1.2015 | **Pancreatit** | **Pancreatit** |  | Kirurgi: 9 – 11 + 12.30 – 14.30 = 1 læge + 1 backup  Akutlæge: 9 – 11 + 12.30 – 14.30 = 1 læge + 1 backup  Sk.st sgpl: 9 - 11 = 2 sgpl  Akut 1: 12.30 - 14.30 = 2 sgpl | |  |
| **3** | Onsdag 14.1.2015 | **KOL** |  | **KOL** | Medicin: 9 – 11 + 12.30 – 14.30 = 1 læge + 1 backup  Akutlæge: 9 – 11 + 12.30 – 14.30 = 1 læge + 1 backup  Sk.st sgpl: 9 – 11 = 2 sgpl  Akut 2: 12.30 – 14.30 = 2 sgpl | |  |
|  | Torsdag 15.1.2015 | **Sepsis** | **Sepsis** |  | Kirurgi: 9 – 11 + 12.30 – 14.30 = 1 læge + 1 backup  Akutlæge: 9 – 11 + 12.30 – 14.30 = 1 læge + 1 backup  Sk.st sgpl: 9 - 11 = 2 sgpl  Akut 1: 12.30 - 14.30 = 2 sgpl | |  |
| **4** | Onsdag 21.1.2015 | **Sepsis** |  | **Sepsis** | Medicin: 9 – 11 + 12.30 – 14.30 = 1 læge + 1 backup  Akutlæge: 9 – 11 + 12.30 – 14.30 = 1 læge + 1 backup  Sk.st sgpl: 9 – 11 = 2 sgpl  Akut 2: 12.30 – 14.30 = 2 sgpl | |  |
|  | Torsdag 22.1.2015 | **Pancreatit** | **Pancreatit** |  | Kirurgi: 9 – 11 + 12.30 – 14.30 = 1 læge + 1 backup  Akutlæge: 9 – 11 + 12.30 – 14.30 = 1 læge + 1 backup  Sk.st sgpl: 9 - 11 = 2 sgpl  Akut 1: 12.30 - 14.30 = 2 sgpl | |  |
| **5** | Onsdag 28.1.2015 | **KOL** |  | **KOL** | Medicin: 9 – 11 + 12.30 – 14.30 = 1 læge + 1 backup  Akutlæge: 9 – 11 + 12.30 – 14.30 = 1 læge + 1 backup  Sk.st sgpl: 9 – 11 = 2 sgpl  Akut 2: 12.30 – 14.30 = 2 sgpl | |  |
|  | Torsdag 29.1.2015 | **KOL** | **KOL** |  | Kirurgi: 9 – 11 + 12.30 – 14.30 = 1 læge + 1 backup  Akutlæge: 9 – 11 + 12.30 – 14.30 = 1 læge + 1 backup  Sk.st sgpl: 9 - 11 = 2 sgpl  Akut 1: 12.30 - 14.30 = 2 sgpl | |  |
| **6** | Onsdag 4.2.2015 | **Sepsis** |  | **Sepsis** | Medicin: 9 – 11 + 12.30 – 14.30 = 1 læge + 1 backup  Akutlæge: 9 – 11 + 12.30 – 14.30 = 1 læge + 1 backup  Sk.st sgpl: 9 – 11 = 2 sgpl  Akut 2: 12.30 – 14.30 = 2 sgpl | |  |
|  | Torsdag 5.2.2015 | **Pancreatit** | **Pancreatit** |  | Kirurgi: 9 – 11 + 12.30 – 14.30 = 1 læge + 1 backup  Akutlæge: 9 – 11 + 12.30 – 14.30 = 1 læge + 1 backup  Sk.st sgpl: 9 - 11 = 2 sgpl  Akut 1: 12.30 - 14.30 = 2 sgpl | |  |
|  | | | | | | |  |
