# Supplementary material for: Designing in situ simulation in the emergency department: evaluating safety attitudes amongst physicians and nurses
Source: Adv Simul (Lond). 2017 Feb 8;2:4. doi: 10.1186/s41077-017-0037-2 (PMC5806390; doi:10.1186/s41077-017-0037-2)
Supplement: Supplementary file 5 — In situ plan. (PDF 1566 kb) [file 41077_2017_37_MOESM5_ESM.pdf]

IN SITU PLAN!

8.00 opsætning

Modtage figuranter
- pr. os
- sminkes (lilla tush på fingre)
- tøj?

— " — instruktør
- pr. os
- lokaler

Finde koordinater på Akut 1/2

Adviser Akutlæge, mediciner, kirurg → seddel til lommen! Find vagtplan

SIM VIDEO

9.00 SAQ

9.00 - 9.15 Info om det at simulere, spilleregler; Underskrive samtykke } v/Runner

9.15 - 9.20 BRIEFING: kl 10.05 STOP → OVERLEVERING

PT ER i SENGEN

SCENARIE-TID = 45 min

- SGPL
- AKUTLÆGE
- MEDICINER / KIRURG

OPGAVER: OVERLEV., TRIAGE, TOKS, BL. PR, RTG, MONITORERING, MM

10.05 - 10.10 OVERLEVERING

DEBRIEF + DIKTAFONEN SOM "DELTAGER" PÅ EGEN STOL!

11.00 SAQ

Hvem har meldt pt? Egen læge?
↓ kommer m. Falck. Primære besked på?

→ pt. historie → sgpl.
Akutlæge får historien af runner uden for døren? + af sgpl når akutlæge kaldes → pt. FIND UD AF OM AKUTLÆGEN ALTID HAR HØRT OM PT FØR ANK.

MEDICINER/KIR RINGER TIL AKUT 1/2's KOORD. SGPL.

FORBLIVE i LOKALET = OVERLEVERING VIDEOOPTAGES

OVERLEV!

- HVAD HØRTE DU?
- HVAD VIL DU FORTÆLLE TIL MODTAGENDE SGPL?
- VAR DER DELE AF OVERLEVERINGEN, DER VAR AFGØRENDE FOR DIN UDDELEGERING AF PATIENTEN?
- HVORNÅR FORVENTER DU PATIENTEN?
- HVORDAN SER AFDELINGEN UD LIGE NU - ANTAL PT OG RESSOURCER?

{ HUSK AT INDTALE DATO, TID, STED }

RUNNER LAVER INTERVIEW M. KOORD. SGPL AKUT 1/2
OPTAGES PÅ DIKTAFON
→ MED TIL DEBRIEF
+ TRANSKRIBERES
→ BESKED TIL MODTAGENDE SGPL.

FROKOST
- OPRYD
- OPSÆTNING AKUT 1/2
- TRANSKRIBERING
- OPDATERE EPJ
- DE-DEBRIEF
- SPISE

11 - 12.30

12.30 SAQ

12.30 - 12.45 Info om det at simulere, spilleregler; Underskrive samtykke } v/Runner

12.45 - 12.50 BRIEFING: kl 13.35 STOP → OVERLEVERING

RUNNER CITERER ORDRET DET TRANSKRIB. "PT RULLER IND!"

SCENARIETID = 45 min

- SGPL
- AKUTLÆGE
- MEDICINER / KIRURG

OPGAVER: OVERLEV, TOKS, OPFØLG, MONITOR, PLEJE, MM

13.35 - 13.40 OVERLEVERING?

13.40 - 14.30 DEBRIEF + evt diktafon som "deltager" på egen stol!

14.30 SAQ

overlevere mediciner/kirurg → med/kir + Akutlæge → akutlæge
Samme akutlæge, med, kir i begge scenarier
7a ⇒ ↓ SAQ, ↑ belastning på samme læge

Skal vi indbygge en overlevering, tx → modt. sgpl i AV? Bindes af RUNNER. Men hvem "modtager" overleveringen? Vi skal "bruge" en sgpl i "den anden ende"

OPRYD
FARVEL OG PEPT TAK
DE DEBRIEF
- INSTRUKTØR
- FIGURANTER } v/Runner.

Runner har flere opgaver ⇒ ej så som instruktør!

TEKNIK OG SAGER!

IPAD = MONITOR

SCREEN DUMPS
- Edok
- Dept triage
- TOKS

EPJ OVESYSTEM
- PT OPLYSNINGER
- BL. PR?
- RTG?
- DIKTATER

ATRAP ARM
— BUFRE

TELEFONER
- AKUTLÆGE
- KOORD. SGPL AKUT 1/2
- MEDICINER
- KIRURG
- LAB } = Anders
- RTG } SVAR KORT; et eller andet

DIKTAFON

PC / PRINTER
→ ADGANG translitterering

Tilføje journal oplysninger i frokostpausen!

Niv BHL i ny SKSTUE + AKUT 1/2?

placebomedicin / Naci "fisk" m. påtapet ampul
patient labels

Bestille "pr. os"
- kaffe, the, chokolade kiks
- Frokost
- vand

— HVOR, HVEM?
Anders EAN
Falck journal skaffes

SAQ HVORNÅR?

Seddel m A-gas bl. pr. journal-notat rtg

lost

www.deptriage.dk
adgang: DEPT
kode: DEPT

OPMÆRKSOMHEDS TING! = FIND UD AF CP
