# Supplementary material for: Designing in situ simulation in the emergency department: evaluating safety attitudes amongst physicians and nurses
Source: Adv Simul (Lond). 2017 Feb 8;2:4. doi: 10.1186/s41077-017-0037-2 (PMC5806390; doi:10.1186/s41077-017-0037-2)
Supplement: Supplementary file 6 — Urosepsis scenario. (DOC 83 kb) [file 41077_2017_37_MOESM6_ESM.doc]

| **Scenario** | **Urosepsis** | |
| --- | --- | --- |
| **Participants** | Emergency department (ED) Nurses, EM physicians, medical physician in charge | |
| **Participant prerequisites** | Professional qualifications: Nurses, residents, and physicians, employed by/or associated with the Emergency Department.  Other: Having signed the consent form and completed the questionnaire prior to attending the simulation. | |
| **Duration** | Questionnaire + Briefing: 25 min Scenario: 40 min Debriefing: 40 min | |
| **Learning objectives** | Technical skills   1. Treatment of urosepsis according to existing guidelines | Non-technical skills   1. Apply teamwork principles; such as cooperation, leadership, communication, resource utilization, and prioritization 2. Use safe communication as SBAR and closed loops during treatment and handover 3. Use a structured approach, such as ABCDE during assessment and treatment. |
| **Case** | 27 year-old male with a fever and minor left-side flank pain.  The patient has sepsis. He is complaining. Is easily confused (due to dehydration/malaise)  There is no greater development in the scenario. Responsive to fluid (minor improvement – steady state).  After approximately 35 minutes, the patient must be transferred to the medical ward. The medical physician must arrange and ensure transfer and do the handover to the coordinating ward nurse by telephone. | |
| **Setup**  **ED Room**  **Medicine room**  **Debriefing room**  **Other** | Simulated patient (SP), instructed with the information below  ED room in situ with usual equipment (bed, monitor, etc.).  Computer with access to practice-Electronic Patient Journal (SP journal and data has been established. Arterial blood gas analysis are available on paper)  Video cameras + audio (extra battery + SD card)  2 iPads + charger, 1 fixed to monitor the ED room, one used as a remote control. Simmon installed.  Board with phone numbers:   - EM physician - Medical physician in charge - Lab (answered by the secondary scenario facilitator) - X-ray (answer by the secondary scenario facilitator: “We have time for the patient in xx minutes (xx = number of minutes until scenario is finished) - Coordinating ward nurse   Ongoing simulation sign on the door  Part task trainer: Lower abdomen/Catheterization trainer  Social Security Card  Boxed and marked simulation: Pre-filled syringes NaCl labelled and used as universal medicine. However, antibiotics are mixed.  A clock visible to the facilitator  Chairs to 5-6  Camcorder + audio + charger / battery + SD card  Laptop + charger that can play video sequences from the scenario  Coffee and water for SP, facilitator and secondary scenario facilitator   - Interview Guide + voice recorder to record the handovers - Extra batteries - SAQ-DK Questionnaires - Consent forms - Pens/pencils - Make up/moulage kit for the SP - Lunch for facilitator and secondary scenario facilitator | |
| **Scenario**  **Paramedic handover** | 27 year-old male, previously well  **Paramedic**  *3 days ago he went to his GP with painful and burning urination and a slight fever. Urine was tested positive and treatment for cystitis was initiated.*  *For the last 12 hours, there has been increasing pain over the bladder and fever. Friend visited and telephoned the doctor who inserted the patient. Pt.had chills and fever. Seems a little confused and tired.*  ***ABCDE***  *RR: 24, SaO^2^ = 99% on 12L oxygen, Puls = 115, BP = 95/70, Skin feels warm and damp, sweating, PVK has been inserted.*  **Arterial-blood gas 1 Arterial-blood gas 2**  pH: 7.32 pH: 7.32  PaO2: 11.2 PaO2: 11.2  PaCO2: 4.3 PaCo2: 4.3  Lactate: 4.5 Lactate: 4.5  HCO3: 22.0 HCO3: 22  Base Excess: - 2.0 Base Excess: - 2.0  BG: 8.2 BG: 8.2  NaCl: 132.5 NaCl: 135.4  K: 4.5 K: 4.5  Ca: 1.01 Ca: 1.01  SaO2: 99 SaO2: 99  HGB: 8.2 HGB: 7.7  Hct©: 44.5 Hct©: 44.5  Other clinical examination results:  Ultrasound = Left sided hydronephrosis  If CT abdomen is requested the patient will have to be transferred out of the ED.  CT abdomen answer: Slightly dilated renal pelvis on the left side. Otherwise nothing abnormal. | |

| **Expected development and treatment during the scenario**  **Patient’s development during scenario** | Paramedic handover  Admission to the ward  Triage and measurement of vital parameters  Oxygen (PVK inserted prehospital) – iv. fluid .  Examination for neck / back stiffness: NO neck / back stiffness found  Level of consciousness examination  Arterial-blood gas sample and analysis  Thorax x-ray: Nothing abnormal  Bladder catheter  iv. antibiotics  Call for the EM Physician (within 15 minutes)  Presumed prescriptions:  iv.:Tazocin (piperacillin and tazobactam) 4 g x 3 or Selexid (pivmecillinam) 1g x 3 iv.  Pain relief: Paracetamol  Start of iv fluid (4-6 litres the first 24 hours)  Arterial-blood gas sample when possible and again after an hour  Urine test strip + urine culture and resistance  Blood for culture and resistance x 2  Bloodsamples: (leukocytes, diffrential count, platelets, CRP, Hb, erythrocytes, MCV, MCHC, erythrocyte fraction, BG, potassium, sodium, creatinine, urea, amylase, ALT, alkaline phosphatase, LDH, Bilirubin, APTT, PP / INR)  Ultrasound /CT abdomen/ thorax x-ray  Bladder catheter (on task-trainer)  Patient’s vital signs stay more or less constant with minor response to fluid bolus.  Pt must be transferred to the ward. While waiting for transport, CT abdomen can be performed (if requested). No matter the development of the scenario it will have to end after 40 minutes.  Handover must be given to the coordinating ward nurse. |
| --- | --- |

| **Operator Information ABCDE** |  | **Initially** | **After fluid administration: 1-2L** |
| --- | --- | --- | --- |
|  | A | Patent | Patent |
|  | B | RR 24  PaO2 99% on 12L oxygen (Hudson mask with reservoir from the ambulance)  St. P: nothing abnormal | Unchanged |
|  | C | HR: 125  BP: 95/70  Sinus rhythm  St. C: nothing abnormal  Pale, warm and dry  Patient has PVK | HR: 110  BP: 100/70 |
|  | D | Patient has eyes closed. Opens when spoken to.  Pupils are 4mm, normal response to light. Patient is alert and oriented , but slightly confused  BG: 8,2  GCS 13-14 – (1 point for eyes, 1-2 point for verbal response). | GCS: 13-14 |
|  | E | Temperature 39.1^o^C  Urine test strip: blood ++, leucocyte +++, nitrite +, protein ++ (clear no smell). Sore on kidney percussion and palpation. No neck / back stiffness.  VAS: 3-7 (Worst when urinating)  Not previous hospitalised. No known allergies.  Normal stools. No trauma to flank, back or stomach. | VAS 3  Temperature 39.2^o^C |
|  | Test results:  Ultrasound = Left sided hydro-nephrosis  CT abdomen: slightly dilated renal pelvis on the left side. Otherwise nothing Thorax x-ray: nothing abnormal | | |
| **Simulated patient information** | You are 27 years and healthy. You work in the grocery store in the warehouse, and living by yourself.  3 days ago you went to your GP due to symptoms of bladder infection and fever. Urine test strip were positive and you were given antibiotics due to suspicion of cystitis.  You've had a friend sleep over last night. And have become quite ill during the night. Your friend has attended to you several times. This morning, you were miserable. Your friend was uneasy to let you alone and contacted your GP, who admitted you to the hospital. (Your friend was going to Copenhagen for funeral).  You are having pain over the bladder - lower abdomen, burning and pain when urinating. It is as if there are spasms of the bladder when urinating. You are only able to urinate very little at the time (it’s painful). Urine is concentrated, but without blood.  You feel, like you've got the flu; chills, and muscle soreness all over.  Not previously hospitalized. No known allergies.  You have normal bowel movements. No trauma to the flank, back or stomach.  On arrival at the emergency department you act as below:  VAS 3-7 (worst when urinating)  You have a sensations of fever, you have chills and feel tired  You are breathing fast (RR 24).  Your condition does not change significantly during the scenario.  **Moulage information:**  PKV attached to the arm with gauze + infusion of Saline (NaCl) + drain device. Hudson mask with reservoir.  Patient is a little pale with red cheeks + day-old makeup  Clammy Sweating peripheral and head  Part-task-trainer used for catheterisation under the bed.  Oxygen mask (Hudson mask with reservoir) | | |
| **Participant information** | Nurse 1 and 2:  You are about to receive and admit a patient. You’ll get further information from the patient, paramedics and scenario facilitator if needed.  The ED coordinator has just given the following message: Paramedics are bringing in a young patient with stomachache. Patient is a little confused. Apparently ABC stable, though slightly low BT, elevated pulse and RR.  EM physician/resident:  You will be called when the nurse finds it necessary. You know from the coordinators who have read the paramedics’ admission message that they are bring in a young patient with stomachache. Patient is a little confused. Currently, Apparently ABC stable, though slightly low BT, elevated pulse and RR.  Medical physician in charge:  You will be called at some point during the scenario. You have no information about the patient yet. You can call a senior consultant if necessary. | | |
